# Will avilamycin convert ziracine into zerocine?

**DOI:** 10.3201/eid0703.010336

**Published:** 2001

**Authors:** T R Shryock


sequencing, was also used to confirm the base calls of these 100 SNPs. The visual inspection of the electropherograms and the sequencing independent method were in good agreement and indicated that 80 (91%) of 88 successful assays of the nucleotide differences were genuine.

Since our initial report, we have improved our methods for overlaying the annotation of open reading frame coordinates onto our analysis of the coordinates of nucleotide substitutions. Approximately 7% of the genome is noncoding, and approximately 15% of the substitutions are in these regions.

Dr. Musser is correct in pointing out that the substitution frequency expressed in Fraser et al. (5), based on our preliminary annotation of our *M. tuberculosis* sequence data, is not an equivalent comparison to the synonymous substitution frequency derived by his method of sequencing a select set of genes over a wide range of *M. tuberculosis* strains. He uses the methods of Li et al. (6), among the most widely accepted, for the calculation of nucleotide substitution frequencies and derives a $D_s$ value of <0.01 synonymous substitutions per 100 synonymous sites. Our preliminary data presented the frequency of total nucleotide substitutions at all positions (coding [synonymous and nonsynonymous] and noncoding) of the two recently sequenced strains, H37Rv and CDC1551. Our manuscript in preparation comparing the two *M. tuberculosis* strains will contain an analysis of synonymous substitutions. However, while Dr. Musser compared a select group of genes over perhaps several hundred strains, our frequency will be based on a genome-wide comparison between two strains.

**Robert Fleischmann**
The Institute for Genomic Research
Rockville, Maryland, USA

### References

1. Cole ST, Brosch R, Parkhill J, Garnier T, Churcher C, Harris D, et al. Deciphering the biology of *Mycobacterium tuberculosis* from the complete genome sequence. Nature 1998;393:537-44.
2. Delcher AL, Kasif S, Fleischmann RD, Peterson J, White O, Salzberg SL. Alignment of whole genomes. Nucleic Acids Res 1999;27:2369-76.
3. Weinstock GM. Genomics and bacterial pathogenesis. Emerg Infect Dis 2000;6:496-504.
4. Fleischmann RD, Adams MD, White O, Clayton RA, Kirkness EF, Kerlavage AR, et al. Whole-genome random sequencing and assembly of *Haemophilus influenzae* Rd. Science 1995;269:496-512.
5. Fraser CM, Eisen J, Fleischmann RD, Ketchum KA, Peterson S. Comparative genomics and understanding of microbial biology. Emerg Infect Dis 2000;6:505-12.
6. Li WH, Wu CI, Luo CC. A new method for estimating synonymous and nonsynonymous rates of nucleotide substitution considering the relative likelihood of nucleotide and codon changes. Mol Biol Evol 2000;2:150-512.

### Will Avilamycin Convert Ziracine into Zerocine?

**To the Editor:** Dr. Courvalin urges that avilamycin be prospectively banned as an antibiotic growth promoter to prevent the development of bacteria cross-resistant to the potential human-use product evernimicin (1). Elanco Animal Health, the manufacturer of avilamycin, would like to clarify the situation with respect to avilamycin and everninomicin. It should be noted that there is incomplete cross-resistance in that enterococci resistant to avilamycin exhibit only decreased susceptibility, not complete resistance, to everninomicin (2). Dr. Courvalin's recommendation has become moot, since Schering-Plough has discontinued clinical development of Ziracin, as announced in early May 2000, "because the balance between efficacy and safety did not justify further development of the product" (http://www.sch-plough.com/news/research/2000/050500.html). Thus, avilamycin actually remains in compliance with the Swann principles. In addition, the Scientific Committee on Animal Nutrition, which advises the European Union Commission, released its assessment of the potential impact from cross-resistance in late April 2000 (http://www.europa.eu.int/comm/food/fs/sc/scan/out48_en.pdf) and concluded that, although transfer of resistant bacteria–and presumably resistance genes–from animal to human bacteria is possible, the magnitude of the transfer with avilamycin resistance was not possible to predict. In part, this conclusion reflected the early developmental status of Ziracin and a few reports of clinical experience. An extensive survey of Ziracin showed that 100% of 4,208 enterococcal isolates from patients in 27 European countries were susceptible (3). Another survey of Ziracin showed that 99.5%-100% of 6,030 isolates of methicillin-resistant *Staphylococcus aureus*/*epidermidis*, enterococci, streptococci, and pneumococci from 33 laboratories around the world were susceptible (4). Avilamycin has been used in animal production in many of the countries from which these clinical isolates originated. To fairly balance a preemptive precautionary action against a currently marketed animal use product and a human clinical candidate, the World Health Organization Global Principles recommended that such an action be initiated only when the human clinical candidate dossier is submitted for regulatory approval, to ensure that the candidate will indeed enter the marketplace. (Use of antimicrobial growth promoters that belong to classes of antimicrobial agents used or submitted for approval in humans and animals should be terminated or rapidly phased out in the absence of risk-based evaluations.) [http://www.who.int/emc/diseases/zoo/who_global_principles.html#Purpose]). This recommendation also acknowledges, in accordance with the Swann Principles, that antimicrobial agents intended for nonhuman use can be used in animal production. The modification by the pharmaceutical industry of older classes of antimicrobials for human clinical use, with counterparts previously developed by animal health companies for use as growth promoters, has become common. Dr. Aarestrup of the Danish Veterinary Laboratory commented that "it will be necessary in the future to either totally avoid the use of antimicrobials for growth promotion or, once antimicrobials have been approved for growth promotion, to reserve these classes for growth promotion and search for therapeutic options among other classes" (2). With respect to avilamycin, this latter option is the better one, now that everninomicin (and perhaps the entire orthosomycin class by extension) has been demonstrated to be unsafe for parenteral or injectable use in humans, because it allows animal producers to use a product that poses no resistance threat to public health. Finally, other unique antibiotics for treatment of serious gram-positive infections in humans (with no animal use counterparts) are in the pharmaceutical pipeline (e.g., LY333328 and daptomycin) or have recently been approved (e.g., linezolid). We hope that a fair balance can be achieved by the human medical and the animal health and production communities with regard to the types of antimicrobial agents that can be used in each sector.

**Thomas R. Shryock**
Elanco Animal Health, Greenfield, Indiana, USA

### References

1. Courvalin P. Will avilamycin convert ziracine into zerocine? Emerg Infect Dis 2000;6:558.
2. Aarestrup FM. Association between decreased susceptibility to a new antibiotic for treatment of human disease, everninomicin (SCH 27899), and resistance to an antibiotic used for growth promotion in animals, avilamycin. Microb Drug Resist 1998;4:137-41.
3. Schouten MA, Voss A, Hoogkamp-Korstanje JAA, and the European VRE Study Group. Antimicrobial susceptibility patterns of enterococci causing infections in Europe. Antimicrob Agents Chemother 1999;43:2542-6.
4. Jones RN, Hare RS, Sabatelli FJ, Ziracin Susceptibility Testing Group. In vitro gram-positive antimicrobial activity of everniminicin (SCH 27899), a novel oligosaccharide, compared with other antimicrobials: a multicentre international trial. J Antimicrob Chemother 2001;47:15-25.

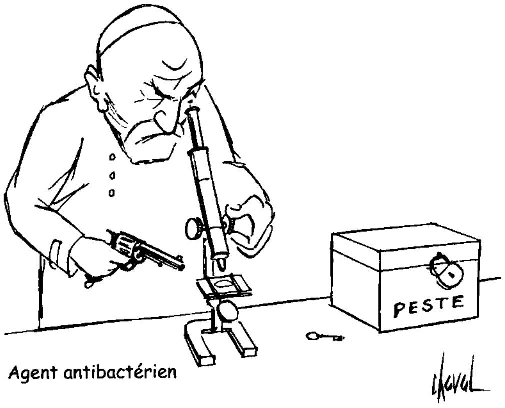

Cartoon from poster of annual meeting of Société Française de Microbiologie, Section des Agents Antimicrobiens. Used with permission and courtesy of P. Courvalin.

### The Antibiotic Food-Chain Gang

**To the Editor:** In his reply to my letter (1), Dr. Shryock states that use of the growth promoter avilamycin, which confers cross-resistance to other members of the everninomycin class of drugs, was in compliance with the Swann principles. The Swann report, issued in 1969, recommends that antibiotics used to treat infections in humans not be used as animal-food additives (2). The combined efforts of many scientists were needed to bring about the 1999 ban in Europe of spiramycin, tylosin, virginiamycin, and bacitracin, each of which confers resistance to antibiotics used in clinical settings. It appears that more than 30 years was necessary for the animal-food industry to act in accordance with the Swann report.

The reasoning in terms of drug structures can be misleading. The implication is that drugs that are chemically closely related have the same target of action and are therefore subject to cross-resistance, and vice versa. For example, because it has an unusual structure, apramycin (a 4-substituted-2-deoxystreptamin) was used exclusively in animals in the hope that it would not be recognized by any of the known aminoglycoside-modifying enzymes (3). However, enterobacteria of animal origin were resistant to apramycin by synthesis of a plasmid-mediated 3-*N*-aminoglycoside acetyltransferase type IV, which also confers resistance to gentamicin (4). Following spread in animal strains (5), the plasmid was later found in clinical isolates from hospitalized patients (6).

The use of antibiotics in general should be based on the mechanisms of resistance in bacteria, rather than on their chemical makeup. In particular, the concept that resistance was a class phenomenon rapidly lost favor because of the extension of the concept of cross-resistance and the increased occurrence of co-resistance.

In classical cross-resistance, a single biochemical mechanism confers resistance to a single class of drugs: use of a given antibiotic can select resistance to other members of the group but not to drugs belonging to other classes. However, cross-resistance between drug classes can occur by two mechanisms: overlapping targets and drug efflux. An example of target overlap is provided by the macrolides, lincosamides, and streptogramins (MLS), which are chemically distantly related. However, constitutive methylation of a single adenine residue in ribosomal RNA confers high-level resistance to the three classes of antibiotics. This resistance phenotype is due to the fact that all these antibiotics have overlapping targets on the ribosome (7). Active efflux of the drugs outside bacteria has recently been recognized as a common resistance mechanism (8,9). This energy-dependent export confers low-level resistance to a wide variety of antibiotics. The broad substrate specificities of the pumps account for decreased susceptibility to beta-lactams, aminoglycosides, tetracyclines, chloramphenicol, trimethoprim, sulfonamides, fluoroquinolones, and MLS, among others (9).

In contrast to cross-resistance, co-resistance is due to the presence in the same host of several mechanisms, each conferring resistance to a given class of drugs. In addition, the corresponding genes are often adjacent (physically linked) and expressed in a coordinated fashion. One of the most efficient system of this type is represented by the integrons (10) first described in gram-negative bacilli (11,12) and more recently found in gram-positive bacteria (13). Because of the genetic organization resulting in co-expression of the various genes, use of any antibiotic that is a substrate for one of the resistance mechanism will co-select for resistance to the others and thus for maintenance of the entire gene set. Since cross-resistance means cross-selection and co-resistance implies co-selection, the use of any antimicrobial agent is de facto rendered inadequate as a growth promoter.

I also disagree with the notion that because a member of an antibiotic class has been misused as a growth promoter the class should not be used in the future for human

